# Antibacterial, antifungal and cytotoxic activities of amblyone isolated from *Amorphophallus campanulatus*

**DOI:** 10.4103/0253-7613.40489

**Published:** 2008

**Authors:** Alam Khan, Moizur Rahman, M.S. Islam

**Affiliations:** Department of Pharmacy, University of Rajshahi, Rajshahi 6205, Bangladesh; 1 Department of Animal Husbandry and Veterinary Science, University of Rajshahi, Rajshahi 6205, Bangladesh

**Keywords:** Gram-negative, Gram-positive, MIC, triterpenoid

## Abstract

**Objective:**

To assess the *in vitro* antibacterial, antifungal and cytotoxic activities of amblyone, a triterpenoid isolated from *Amorphophallus campanulatus* (Roxb).

**Methods:**

Disc diffusion technique was used for *in vitro* antibacterial and antifungal screening. Cytotoxicity was determined against brine shrimp nauplii. In addition, minimum inhibitory concentration (MIC) was determined using serial dilution technique to determine the antibacterial potency.

**Results:**

Large zones of inhibition were observed in disc diffusion antibacterial screening against four Gram-positive bacteria (*Bacillus subtilis, Bacillus megaterium, Staphylococcus aureus* and *Streptococcus pyogenes*) and six Gram-negative bacteria (*Escherichia coli, Shigella dysenteriae, Shigella sonnei, Shigella flexneri, Pseudomonas aeruginosa* and *Salmonella typhi*). The MIC values against these bacteria ranged from 8 to 64 μg/ml. In antifungal screening, the compound showed small zones of inhibition against *Aspergillus flavus, Aspergillus niger* and *Rhizopus aryzae*. *Candida albicans* was resistant against the compound. In the cytotoxicity determination, LC_50_ of the compound against brine shrimp nauplii was 13.25 μg/ml.

**Conclusions:**

These results suggest that the compound has good antibacterial activity against the tested bacteria, moderate cytotoxicity against brine shrimp nauplii and insignificant antifungal activity against the tested fungi.

## Introduction

The frequency of life-threatening infections caused by pathogenic microorganisms has increased worldwide and is becoming an important cause of morbidity and mortality in immunocompromised patients in developing countries.[1] Although a large number of antimicrobial agents have been discovered, pathogenic microorganisms are constantly developing resistance to these agents.[1] In recent years, attempts have been made to investigate the indigenous drugs against infectious diseases.[2] This may help to develop safer antimicrobial drugs.[2]

**Figure 1:**
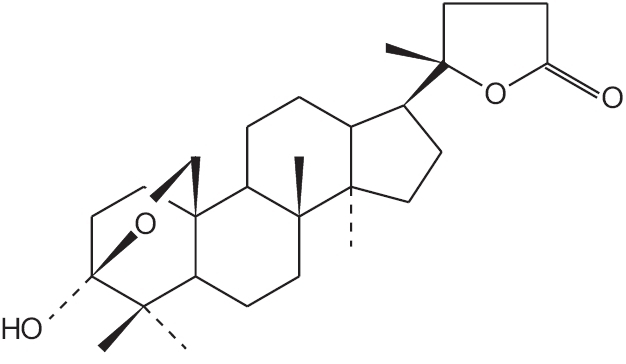
Structure of amblyone

*Amorphophallus campanulatus* (Roxb.) Bl. (family: Araceae), locally known as Ol Kachu, is a perennial herb with rounded tuberous root stock (corm). The plant is widely distributed in Bangladesh, India and Africa.[3–5] The tuberous roots of the plant have been used traditionally for the treatment of piles, abdominal pain, tumours, enlargement of spleen, asthma and rheumatism.[3–5] The tuberous roots of the plant have also been reported to possess tonic, stomachic and appetizer properties.[4,5] Previously, we have reported the possible antibacterial, antifungal and cytotoxic activities of tuberous roots of *Amorphophallus campanulatus*.[24] The present study was conducted to determine the antibacterial, antifungal and cytotoxic activities of amblyone, a triterpenoid isolated from tuberous roots of the plant (Fig 1).

## Materials and Methods

### Plant material

The tuberous roots of *Amorphophallus campanulatus* was collected during January 2004 from Katakhali area of Rajshahi district of Bangladesh and identified by Prof. A.T.M. Naderuzzaman, Department of Botany, University of Rajshahi, where its voucher specimen (No. AC9642) was deposited. The tuberous roots were cut, air-dried and ground into powder.

### Plant material extraction and fractionation

Powdered dried roots (600 g) of the plant were extracted (cold) with ethanol (4 l) in flat bottom glass containers, through occasional shaking and stirring for 10 days.[6] The whole extract was filtered and the solvent was evaporated to dryness *in vacuo* with rotary evaporator at 40-50°C to afford a blackish green mass (34 g), which was further extracted with petroleum ether (3 × 50 ml), chloroform (3 × 50 ml) and methanol (3 × 50 ml) and to afford petroleum ether (17 g), chloroform (8 g) and methanol (7 g) fractions, respectively.[7] The preliminary phytochemical screening of different fractions was carried out by chemical tests and thin layer chromatographic methods.[8]

### Isolation of compound

The petroleum ether soluble fraction (3 g) was subjected to column chromatography using n-hexane, chloroform and methanol of increasing polarity. Column chromatography fractions eluting with 80% chloroform in n-hexane to 100% chloroform were subjected to preparative TLC (Silica gel PF_254_) with solvent system ethyl acetate:cyclohexane (3:1) to yield compound 1 (27.6 mg). Its structure was confirmed on the basis of spectroscopic methods [IR, liquid chromatography/electrospray-mass spectroscopy (LC/ES-MS), ^1^H and ^13^C NMR including JMOD, COSY, NOESY, HMBC, HSQC]. The LC/ES-MS in the positive ion mode of 1 showed molecular [M + H]^+^ peak at m/z 431.5 corresponding to a molecular formula of C_27_H_42_O_4_. ^1^H and ^13^C NMR data of compound 1 were in good agreement with ^1^H and ^13^C NMR data of amblyone published in literature.[9]

Antibacterial activity and minimum inhibitory concentration (MIC) were determined against four Gram-positive bacteria (*Bacillus subtilis*, *Bacillus megaterium*, *Staphylococcus aureus* and *Streptococcus pyogenes*) and six Gram-negative bacteria (*Escherichia coli*, *Shigella dysenteriae*, *Shigella sonnei*, *Shigella flexneri*, *Pseudomonas aeruginosa* and *Salmonella typhi*). Antifungal screening was carried out against four fungi (*Aspergillus flavus*, *Aspergillus niger*, *Candida albicans* and *Rhizopus aryzae*). All these tested organisms were available in the Microbiology Research Laboratory of Pharmacy Department, Rajshahi University, Bangladesh. Cytotoxicity was determined against brine shrimp nauplii that were obtained by hatching brine shrimp eggs (Carolina Biological Supply Company, Burlington, NC, USA) in artificial seawater (3.8% NaCl solution) for 48 h.

### Media

Nutrient agar media (Difco laboratories) pH 7.2, nutrient broth media (Difco laboratories) pH 6.8, Sabouraud dextrose agar media (Biolife Vole Monza) pH 5.6 and artificial seawater (3.8% NaCl solution) pH 8.4 were used for antibacterial screening, MIC determination, antifungal screening and cytotoxicity determination, respectively.

### Antibacterial screening

*In vitro* antibacterial screening was carried out by disc diffusion method,[10,11] which is a qualitative to semi-quantitative test. Briefly, 20 ml quantities of nutrient agar were plated in Petri dish with 0.1 ml of a 10^−2^ dilution of each bacterial culture (18 h old). Filter paper discs (6 mm in diameter) impregnated with various concentration of amblyone were placed on test organism-seeded plates. Methanol was used to dissolve the compound and was completely evaporated before application on test organism-seeded plates. Blank disc impregnated with solvent methanol followed by drying off was used as negative control. The activity was determined after 18 h of incubation at 37°C. The diameters of zone of inhibition produced by the amblyone were then compared with the standard antibiotic kanamycin 30 μg/disc. Each sample was used in triplicate for the determination of antibacterial activity.

### Minimum inhibitory concentration determination

Serial tube dilution technique[12,13] was used to determine MIC of the compound against these bacteria. Amblyone (1.024 mg) was dissolved in 2-ml nutrient broth media (three drops of Tween 80 added to facilitate dissolution) to obtain a stock solution having concentration of 512 μg/ml. The serial dilution technique was used to obtain 256-2 μg/ml dilutions. One drop (0.02 ml) of prepared suspension of organism (10^7^ organism/ml) was added to each broth dilution. These dilutions were incubated at 37°C for 18 h and examined for the growth. The MIC of amblyone was taken as the lowest concentration that showed no growth. Growth was observed in those tubes where the concentration of the amblyone was below the inhibitory level and the broth medium was turbid. The nutrient broth media with three drops of Tween 80 and kanamycin were used as negative and positive control, respectively.

### Antifungal screening

This was also carried out by disc diffusion method.[10,11] In this method, 20 ml of Sabouraud dextrose was plated in Petri dish with 0.2 ml of a 10^−2^ dilution of each fungal culture (10 h old). The activity was determined after 72 h of incubation at 30°C. The diameter of zone of inhibition produced by the amblyone was then compared with the standard antibiotic nystatin 30 μg/disc. Each sample was used in triplicate for the determination of antifungal activity.

### Cytotoxicity assay

The cytotoxicity assay was performed on brine shrimp nauplii using Mayer method.[14,15] Dissolution of compound was performed in artificial seawater using DMSO. Each 5 ml solution of different concentrations (0.5, 1, 2, 5, 10, 20 and 40 μg/ml) of the compound was taken in different vials where brine shrimp nauplii were placed and observed for mortality for 24 h. The resulting data were transformed to probit analysis[16] for the determination of LC_50_ values of the compound. Artificial seawater medium containing DMSO was used as control. Gallic acid and vincristine sulphate were used as standards in this assay.

## Results

The results of antibacterial activity of amblyone against the test bacteria are presented in Table 1. In comparison to reference standard kanamycin (30 μg/disc), amblyone exhibited significant antibacterial activity at 160 μg/disc. Amblyone showed highest activity against *Bacillus megaterium* and lowest against *Pseudomonas aeruginosa*. The MIC values against Gram-positive and Gram-negative bacteria ranged from 8 to 32 and 16 to 64 μg/ml, respectively [Table 2].

**Table 1:** *In vitro* antibacterial activity of amblyone and kanamycin

*Test organism*	*Strain No.*	*Zone of inhibition (diameter in mm)*		
		
		*Amblyone (80 μg/disc)*	*Amblyone (160 μg/disc)*	*Kanamycin (30 μg/disc)*
Gram-positive				
*Bacillus subtilis*	QL 40	21 ± 1.5	24 ± 1.3	30 ± 2.1
*Bacillus megaterium*	QL 38	22 ± 1.3	26 ± 2.2	28 ± 0.9
*Staphylococcus aureus*	ATCC 259233	19.5 ± 0.8	23 ± 1.5	31 ± 1.4
*Streptococcus pyogenes*	CRL	20.5 ± 1.4	25 ± 2.1	25 ± 1.7
Gram-negative				
*Escherichia coli*	FPFC 1407	15 ± 1.0	20.5 ± 1.1	24 ± 1.4
*Shigella dysenteriae*	AL 35587	18.5 ± 0.8	21.5 ± 0.9	30 ± 2.4
*Shigella sonnei*	AJ 8992	15 ± 1.1	20 ± 1.0	32 ± 1.7
*Shigella flexneri*	AL 30372	16 ± 0.8	21 ± 1.1	28 ± 1.2
*Pseudomonas aeruginosa*	CRL	12 ± 0.7	16 ± 0.9	31 ± 1.7
*Salmonella typhi*	B 56	17 ± 1.1	21 ± 1.2	28 ± 2.1

The control disc used for solvent had no zone of inhibition; hence, this data has not been shown. Data shown in mean ± SEM (*n* = 3)

**Table 2:** Minimum inhibitory concentration of amblyone and kanamycin

*Bacteria*	*MIC values of amblyone (μg/ml)*	*MIC values of kanamycin (μg/ml)*
*Bacillus subtilis*	32	2
*Bacillus megaterium*	8	4
*Staphylococcus aureus*	16	8
*Streptococcus pyogenes*	16	8
*Escherichia coli*	32	8
*Shigella dysenteriae*	16	2
*Shigella sonnei*	32	4
*Shigella flexneri*	16	16
*Pseudomonas aeruginosa*	64	16
*Salmonella typhi*	32	4

The negative control containing solvent had no MIC value; hence, this data has not been shown

Amblyone showed weak antifungal activity against a number of tested fungi [Table 3]. It was observed that amblyone is inactive against *Candida albicans*. In cytotoxicity assay, the LC_50_ value of amblyone was 13.25 μg/ml. The cytotoxicity of amblyone was compared with that of standard gallic acid and vincristine sulphate, whose LC_50_ values were 4.53 and 0.76 μg/ml, respectively [Table 4]. No mortality was found in the control group. An approximate linear correlation was observed between logarithm of concentration and percentage of mortality.

**Table 3:** *In vitro* antifungal activity of amblyone and nystatin

*Test organism*	*Zone of inhibition (diameter in mm)*		

	*Amblyone (80 μg/disc)*	*Amblyone (160 μg/disc)*	*Nystatin (30 μg/disc)*
*Aspergillus flavus*	7 ± 0.7	9 ± 0.8	17 ± 1.3
*Aspergillus niger*	9 ± 0.6	10 ± 1.1	16 ± 1.4
*Candida albicans*	0	0	18 ± 0.9
*Rhizopus aryzae*	8 ± 0.8	10 ± 1.0	15 ± 1.1

The control disc used for solvent had no zone of inhibition; hence, this data has not been shown. Data shown in mean ± SEM (*n* = 3)

**Table 4:** Cytotoxicity of amblyone, gallic acid and vincristine sulphate

*Sample*	*LC_50_ (μg/ml)*	*95% confidence limit (μg/ml)*	*Regression equation*	*X^2^ value*
Amblyone	13.25	9.39-18.69	*Y* = 2.01 + 2.65*X*	1.71
Gallic acid	4.53	3.33-6.15	*Y* = 3.93 + 1.62*X*	1.25
Vincristine sulphate	0.76	0.57-0.82	*Y* = 3.16 + 2.98*X*	0.62

LC_50_ values, confidence limits, regression equations and X^2^ values were calculated by probit analysis

## Discussion

Several plants have been used for the treatment of piles, abdominal pain, tumours, enlarged spleen, asthma and rheumatism.[3–5] Analgesic activity of *Amorphophallus campanulatus* tuber[22] and inhibition of amylase, trypsin and chymotrypsin by *Amorphophallus campanulatus* tuber[23] have also been determined. The antibacterial, antifungal and cytotoxic activities of tuberous roots of *Amorphophallus campanulatus* were also reported.[24] Amblyone was previously isolated from aerial part of *Cleome amblyocarpa* (Capparidaceae)[9] and *Salvia aspera* (Labiatae).[17] Isolation of amblyone from *Amorphophallus campanulatus* and antibacterial, antifungal and cytotoxic studies of amblyone are being for the first time reported how.

Although amblyone showed activity against all tested bacteria, it was better against Gram-positive bacteria than Gram-negative bacteria. Highest activity against *Bacillus megaterium* and lowest activity against *Pseudomonas aeruginosa* was observed and it was supported by serial tube dilution technique. The antifungal activity seems to be clinically insignificant. Cytotoxicity of amblyone against brine shrimp nauplii and its comparison with standard gallic acid and vincristine sulphate indicated a moderate cytotoxicity of amblyone.

Previous reports of antibacterial, antifungal and cytotoxic activities[24] and traditional uses (against tumours and enlarged spleen[3–5]) of tuberous root of the plant support the findings of present studies. Moderate cytotoxicity of amblyone indicate that it can be selected for further cell line assay, because many scientists have shown a correlation between cytotoxicity and activity against the brine shrimp nauplii using extracts or isolated compounds from terrestrial plants.[18–21] However, more studies are needed to elucidate the structure activity relationship of amblyone, toxicological evaluation and to identify other active constituents of the plant *Amorphophallus campanulatus* if any.
